# Supplementary material for: Reputation structure in indirect reciprocity under noisy and private assessment
Source: Sci Rep. 2022 Jun 22;12:10500. doi: 10.1038/s41598-022-14171-4 (PMC9217807; doi:10.1038/s41598-022-14171-4)
Supplement: Supplementary file 1 — Supplementary Information. [file 41598_2022_14171_MOESM1_ESM.pdf]

Supplementary Information of "Reputation structure in indirect reciprocity under noisy and private assessment" by Yuma Fujimoto & Hisashi Ohtsuki

Here, we show a detailed calculation of triples $\{(q_j, \mu_j, \sigma_j)\}_{j=1,\cdots}$ which satisfy Eq. (19).

When the social norm is SJ: In the main text, we have already derived

$$\mu_1 = f_{\rm SJ}^{\rm C}(\mu_1) = f_{\rm SJ}^{\rm D}(\mu_1) = \frac{1}{2}. \tag{S1}$$

It is trivial that the mass q_1 is

$$q_1 = 1. \tag{S2}$$

We now also derive variance σ_1^2. When we substitute μ_1 and q_1 above into Eq. (19), we obtain

$$\begin{aligned} g(p;\mu_1,\sigma_1^2) &= h(\mu_1)g(p; f_{\rm SJ}^{\rm C}(\mu_1), s^2 + (\underbrace{\Delta f_{\rm SJ}^{\rm C}}_{=1-2e_2})^2\sigma_1^2) + (1-h(\mu_1))g(p; f_{\rm SJ}^{\rm D}(\mu_1), s^2 + (\underbrace{\Delta f_{\rm SJ}^{\rm D}}_{=-(1-2e_2)})^2\sigma_1^2) \\ &= h(\mu_1)g(p;\mu_1, s^2 + (1-2e_2)^2\sigma_1^2) + (1-h(\mu_1))g(p;\mu_1, s^2 + (1-2e_2)^2\sigma_1^2) \\ &= g(p;\mu_1, s^2 + (1-2e_2)^2\sigma_1^2). \end{aligned} \tag{S3}$$

By comparing terms of variances between the left and right sides of this equation, we obtain

$$\begin{aligned} &\sigma_1^2 = s^2 + (1-2e_2)^2\sigma_1^2, \\ &\Leftrightarrow \sigma_1^2 = s^2\frac{1}{1-(1-2e_2)^2} = \frac{e_2(1-e_2)}{N}\frac{1}{1-(1-2e_2)^2} = \frac{1}{4N}. \end{aligned} \tag{S4}$$

When the social norm is SS: In the main text, we have already derived

$$\begin{aligned} &\mu_1 = 1-e_2, \\ &\mu_{j+1} = f_{\rm SS}^{\rm D}(\mu_j) \quad (j \geq 1). \end{aligned} \tag{S5}$$

This recurrence relation can be analytically solved as

$$\begin{aligned} &\mu_{j+1} = -(1-2e_2)\mu_j + (1-e_2) \\ &\Leftrightarrow \left(\mu_{j+1} - \frac{1}{2}\right) = -(1-2e_2)\left(\mu_j - \frac{1}{2}\right) \\ &\Leftrightarrow \mu_j = \{-(1-2e_2)\}^{j-1}\left(\mu_1 - \frac{1}{2}\right) + \frac{1}{2} \\ &\qquad = \frac{1-\{-(1-2e_2)\}^j}{2}. \end{aligned} \tag{S6}$$

Now we derive variance σ_j^2 and mass q_j. When we substitute Eq. (S6) into Eq. (19), we obtain

$$\begin{aligned} \sum_{j=1}^{\infty} q_j g(p;\mu_j,\sigma_j^2) &= \sum_{j=1}^{\infty} q_j\{h(\mu_j)g(p; f_{\rm SS}^{\rm C}(\mu_j), s^2 + (\underbrace{\Delta f_{\rm SS}^{\rm C}}_{=0})^2\sigma_j^2) + (1-h(\mu_j))g(p; f_{\rm SS}^{\rm D}(\mu_j), s^2 + (\underbrace{\Delta f_{\rm SS}^{\rm D}}_{=-(1-2e_2)})^2\sigma_j^2)\} \\ &= \sum_{j=1}^{\infty} q_j\{h(\mu_j)g(p;\mu_1,s^2) + (1-h(\mu_j))g(p;\mu_{j+1}, s^2 + (1-2e_2)^2\sigma_j^2)\} \\ &= \left(\sum_{j=1}^{\infty} q_j h(\mu_j)\right) g(p;\mu_1,s^2) \sum_{j=1}^{\infty} q_j(1-h(\mu_j))g(p;\mu_{j+1}, s^2 + (1-2e_2)^2\sigma_j^2). \end{aligned} \tag{S7}$$

By comparing terms between the left and right sides of Eq. (S7), we obtain a recurrence relation for the variances σ_j^2 as

$$\begin{aligned} &\sigma_1^2 = s^2, \\ &\sigma_{j+1}^2 = s^2 + (1-2e_2)^2\sigma_j^2 \quad (j \geq 1). \end{aligned} \tag{S8}$$

This recurrence relation can be solved as

$$\begin{aligned} &\sigma_{j+1}^2 = s^2 + (1-2e_2)^2\sigma_j^2 \\ &\Leftrightarrow \left(\sigma_{j+1}^2 - \frac{s^2}{1-(1-2e_2)^2}\right) = (1-2e_2)^2\left(\sigma_j^2 - \frac{s^2}{1-(1-2e_2)^2}\right) \\ &\Leftrightarrow \sigma_j^2 = (1-2e_2)^{2(j-1)}\left(\sigma_1^2 - \frac{s^2}{1-(1-2e_2)^2}\right) + \frac{s^2}{1-(1-2e_2)^2} \\ &\qquad = s^2\frac{1-(1-2e_2)^{2j}}{1-(1-2e_2)^2} = \frac{e_2(1-e_2)}{N}\frac{1-(1-2e_2)^{2j}}{1-(1-2e_2)^2} = \frac{1-(1-2e_2)^{2j}}{4N}. \end{aligned} \tag{S9}$$

Similarly, by comparing terms between the left and right sides of Eq. (S7), we can obtain a recurrence relation for the masses q_j as

$$\begin{cases} q_1 = \sum_{j=1}^{\infty} q_j h(\mu_j) \\ q_{j+1} = q_j(1-h(\mu_j)). \end{cases} \tag{S10}$$

By using $\sum_{j=1}^{\infty} q_j = 1$, this is solved as

$$q_j = \frac{\prod_{k=1}^{j-1}(1-h(\mu_k))}{\sum_{\ell=1}^{\infty}\prod_{k=1}^{\ell-1}(1-h(\mu_k))}, \tag{S11}$$

where and hereafter we use the convention, $\prod_{k=1}^{0} \cdot = 1$.

When the social norm is SH: In the main text, we have already derived

$$\begin{aligned} &\mu_1 = e_2, \\ &\mu_{j+1} = f_{\mathrm{SH}}^{\mathrm{C}}(\mu_j) \quad (j \geq 1). \end{aligned} \tag{S12}$$

This recurrence relation can be analytically solved as

$$\begin{aligned} &\mu_{j+1} = (1-2e_2)\mu_j + e_2 \\ &\Leftrightarrow \left(\mu_{j+1} - \frac{1}{2}\right) = (1-2e_2)\left(\mu_j - \frac{1}{2}\right) \\ &\Leftrightarrow \mu_j = (1-2e_2)^{j-1}\left(\mu_1 - \frac{1}{2}\right) + \frac{1}{2} \\ &= \frac{1-(1-2e_2)^j}{2}. \end{aligned} \tag{S13}$$

We also derive variance σ_j^2 and mass q_j. When we substitute Eq. (S13) into Eq. (19), we obtain

$$\begin{aligned} \sum_{j=1}^{\infty} q_j g(p;\mu_j,\sigma_j^2) &= \sum_{j=1}^{\infty} q_j\{h(\mu_j)g(p;f_{\mathrm{SH}}^{\mathrm{C}}(\mu_j),s^2 + (\underbrace{\Delta f_{\mathrm{SH}}^{\mathrm{C}}}_{=1-2e_2})^2\sigma_j^2) + (1-h(\mu_j))g(p;f_{\mathrm{SH}}^{\mathrm{D}}(\mu_j),s^2 + (\underbrace{\Delta f_{\mathrm{SH}}^{\mathrm{D}}}_{=0})^2\sigma_j^2)\} \\ &= \sum_{j=1}^{\infty} q_j\{h(\mu_j)g(p;\mu_{j+1},s^2+(1-2e_2)^2\sigma_j^2) + (1-h(\mu_j))g(p;\mu_1,s^2)\} \\ &= \sum_{j=1}^{\infty} q_j h(\mu_j)g(p;\mu_{j+1},s^2+(1-2e_2)^2\sigma_j^2) + \left(\sum_{j=1}^{\infty} q_j(1-h(\mu_j))\right)g(p;\mu_1,s^2). \end{aligned} \tag{S14}$$

By comparing terms between the left and right sides of Eq. (S14), we obtain a recurrence relation for the variances σ_j^2 as

$$\begin{aligned}\sigma_1^2 &= s^2,\\ \sigma_{j+1}^2 &= s^2 + (1-2e_2)^2\sigma_j^2 \quad (j \geq 1).\end{aligned} \tag{S15}$$

Because this recurrence relation is same as Eq. (S8) for the case of $A = \mathrm{SS}$, we obtain

$$\sigma_j^2 = \frac{1-(1-2e_2)^{2j}}{4N}. \tag{S16}$$

Similarly, by comparing terms between the left and right sides of Eq. (S14), we can obtain a recurrence relation for the masses q_j as

$$\begin{cases} q_1 = \sum_{j=1}^{\infty} q_j(1-h(\mu_j)) \\ q_{j+1} = q_j h(\mu_j). \end{cases} \tag{S17}$$

By using $\sum_{j=1}^{\infty} q_j = 1$, this is solved as

$$q_j = \frac{\prod_{k=1}^{j-1} h(\mu_k)}{\sum_{\ell=1}^{\infty}\prod_{k=1}^{\ell-1} h(\mu_k)}. \tag{S18}$$

When the social norm is SC: In the main text, we have already derived

$$\begin{aligned}\mu_1 &= 1-e_2,\\ \mu_2 &= e_2.\end{aligned} \tag{S19}$$

We also derive variance σ_j^2 and mass q_j. When we substitute Eq. (S19) into Eq. (19), we obtain

$$\begin{aligned}\sum_{j=1}^{2} q_j g(p;\mu_j,\sigma_j^2) &= \sum_{j=1}^{2} q_j\{h(\mu_j)g(p;f_{\mathrm{SC}}^{\mathrm{C}}(\mu_j), s^2 + \underbrace{(\Delta f_{\mathrm{SC}}^{\mathrm{C}})}_{=0}{}^2\sigma_j^2) + (1-h(\mu_j))g(p;f_{\mathrm{SC}}^{\mathrm{D}}(\mu_j), s^2 + \underbrace{(\Delta f_{\mathrm{SC}}^{\mathrm{D}})}_{=0}{}^2\sigma_j^2)\}\\ &= \sum_{j=1}^{2} q_j\{h(\mu_j)g(p;\mu_1,s^2) + (1-h(\mu_j))g(p;\mu_2,s^2)\}\\ &= \left(\sum_{j=1}^{2} q_j h(\mu_j)\right) g(p;\mu_1,s^2) + \left(\sum_{j=1}^{2} q_j(1-h(\mu_j))\right) g(p;\mu_2,s^2),\end{aligned} \tag{S20}$$

By comparing terms between the left and right sides of Eq. (S20), we obtain the variances σ_j^2 as

$$\sigma_1^2 = \sigma_2^2 = s^2 = \frac{e_2(1-e_2)}{N} \tag{S21}$$

Similarly, by comparing terms between the left and right sides of Eq. (S20), we obtain the relation that the masses q_1 and q_2 satisfy, as

$$\begin{cases} q_1 = q_1 h(\mu_1) + q_2 h(\mu_2) \\ q_2 = q_1(1-h(\mu_1)) + q_2(1-h(\mu_2)). \end{cases} \tag{S22}$$

By using $q_1 + q_2 = 1$, this is solved as

$$q_1 = q_2 = \frac{1}{2}. \tag{S23}$$
